# Clinical considerations and challenges in TAV-in-TAV procedures

**DOI:** 10.3389/fcvm.2024.1334871

**Published:** 2024-02-19

**Authors:** Ahmad Hayek, Cyril Prieur, Nicolas Dürrleman, Quentin Chatelain, Reda Ibrahim, Anita Asgar, Thomas Modine, Walid Ben Ali

**Affiliations:** ^1^ Structural Heart Intervention Program, Montreal Heart Institute, Montreal, QC, Canada; ^2^ Department of Interventional Cardiology, Hospices Civils de Lyon, Lyon, France; ^3^ Service Médico-Chirurgical: Valvulopathies-Chirurgie Cardiaque-Cardiologie Interventionelle Structurelle, Hôpital Cardiologique de Haut Lévèque, CHU Bordeaux, Bordeaux, France

**Keywords:** coronary obstruction, structural valve deterioration, TAV-in-TAV, TAVR, CT planning

## Abstract

Transcatheter aortic valve replacement (TAVR) has emerged as a viable treatment for aortic valve disease, including low-risk patients. However, as TAVR usage increases, concerns about long-term durability and the potential for addition interventions have arisen. Transcatheter aortic valve (TAV)-in-TAV procedures have shown promise in selected patients in numerous registries, offering a less morbid alternative to TAVR explantation. In this review, the authors aimed to comprehensively review the experience surrounding TAV-in-TAV, summarize available data, discuss pre-procedural planning, highlight associated challenges, emphasize the importance of coronary obstruction assessment and provide insights into the future of this technique.

## Introduction

Transcatheter aortic valve replacement (TAVR) has emerged as a viable alternative to surgery in high-risk (Class I) and intermediate-risk (Class IIa, level of evidence B) patients (1), expanding its indications in recent years (2, 3). Recent data have extended its suitability to low-risk patients, demonstrating favorable and sustained outcomes compared to surgery (4–7). As a result, the number of patients undergoing TAVR has surged significantly (8), and as treatment moves into younger patients, this poses new challenges.

The longevity of patients who undergo TAVR raises concerns about potential valve degeneration and the need for multiple percutaneous interventions over their lifetime. This has given rise to the concept of Transcatheter aortic valve in Transcatheter aortic valve (TAV-in-TAV), introducing several complexities in patient management. Registries and expert consensus efforts have been carried out to assess safety, highlight key considerations, and standardize the pre-procedural work up for these patients.

This paper aims to comprehensively review the currently available data, shedding light on the potentials, challenges, and unmet needs associated with TAV-in-TAV procedures.

### THV types and characteristics

**Figure 1:**
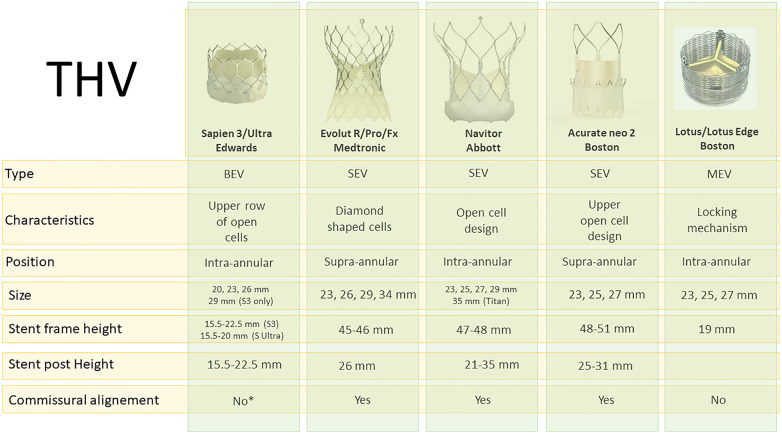
Available THV types and characteristics. *Commissural alignment will be available for Sapien X4. BEV, balloon-expandable valve; MEV, mechanically-expandable valve; SEV, self-expandable valve.

Over the recent years, there has been an evolution of percutaneous valves approved for TAVR. Understanding the characteristics of these valves is crucial for comprehending the challenges posed by TAV-in-TAV procedures. Figure 1 summarizes the features of available and commonly used Transcatheter heart valve (THV)s (9). Essential data include design, leaflet position, and valve frame height. Additionally, knowledge of the skirt height and the degree of expansion of each valve is crucial. A comparison of these data with computed tomography (CT) evaluation of the index valve remains the cornerstone of the TAV-in-TAV intervention. For clarity purposes, we will mainly focus on the Edwards Sapien valves and Medtronic Corevalve/Evolut.

### Pushing the limits of transcatheter aortic valves

The short-term safety and efficacy of Transcatheter aortic valve (TAV) replacement have been extensively studied. As younger patients are now being treated, valve durability has become a critical consideration. To standardize the TAVR field, several societies haves established definitions. The European Association of Percutaneous Cardiovascular Interventions (EAPCI) (10) introduced definitions for bioprosthetic valve dysfunction which encompasses structural valve deterioration (SVD), NSVD, thrombosis, and endocarditis. SVD can result from morphologic and intrinsic valve damage or hemodynamic impairment, while bioprosthetic valve failure (BVF) is linked to severe SVD with clinical signs. In 2018, the Valve in Valve International Data (VIVID) group (11) updated the definition of SVD, outlining four distinctive stages: no immediate changes (stage 0), morphological leaflet abnormalities (stage 1), moderate stenosis or regurgitation (stage 2) or severe (stage 3). More recently, the Valve Academic Research Consortium (VARC-3) Committee (12) refined the EAPCI definitions (Table 1).

**Table 1:** SVD and BVF definitions according to the VARC-3 committee (12).

SVD	Definition	Intrinsic permanent changes to the prosthetic valve, including wear and tear, leaflet disruption, flail leaflet, leaflet fibrosis and/or calcification, or strut fracture or deformation
	Stages	Stage 1: Morphological valve deterioration *Evidence of structural valve deterioration without significant hemodynamic changes*
		Stage 2: Moderate hemodynamic valve deterioration *increase in mean transvalvular gradient* ≥*10 mmHg, resulting in mean gradient* ≥*20 mmHg with concomittant decrease in effective orifice area* ≥*0.3 cm^2^ or* ≥*25% and/or decrease in Doppler velocity index* ≥*0.1 or* ≥*20% compared with echocardiographic assessment performed 1–3 months post-procedure, OR new occurrence or increase of*≥*1 graded of intraprosthetic AR resulting in*≥*moderate AR.*
		Stage 3: Severe hemodynamic valve deterioration *Increase in mean transvalvular gradient*≥*20 mmHg resulting in mean gradient*≥*30 mmHg with concomitant decrease in effective orifice area*≥*0.6 cm^2^ or*≥*50% and/or decrease in Doppler velocity index*≥*0.2% or*≥*40% compared with echocardiographic assessment performed 1–3 months post-procedure, OR new occurrence, or increase of*≥*2 grades,d of intraprosthetic AR resulting in severe AR.*
BVF	Stage 1	Any bioprosthetic valve dysfunction associated with clinically expressive criteria (new-onset or worsening symptoms, LV dilation/hypertrophy/dysfunction, or pulmonary hypertension) or irreversible Stage 3 haemodynamic valve deterioration
	Stage 2	Aortic valve reoperation or re-intervention
	Stage 3	Valve-related death

When considering the long-term durability of TAV using the balloon-expandable (BEV) and self-expandable valve (SEV), data is derived from observational registries. Several studies have reported favorable long-term outcomes with low rates of SVD 5–10 years after the procedure (13–17). In the NOTION trial, SVD was lower after TAVR than after surgical aortic valve replacement (SAVR) (13.9% vs. 28.3% respectively), while the risk of BVF was similar at an 8-year follow-up in low-risk patients (16). These results were recently corroborated in the ESC 2023 update, which showed similar risks of all-cause mortality, stroke and myocardial infarction, higher risk of SVD after SAVR (20.2% vs. 37.7% respectively) and similar risk of BVF at 10 years (18). Sathananthan et al. provided a 10-year follow-up of patients with BEV TAVR, demonstrating a low rate of SVD (6.5%), which is very encouraging in a high-risk population (19). Nevertheless, it's worth noting that SVD rates may be underestimated in these series because serial valve imaging is primarily clinically driven, and isolated morphological valve alterations with no hemodynamic impact may occur. Given the increasing use of TAVR in low-risk patients, SVD is expected to persist despite increased experience, systematic planning, and the introduction of newer valve generations.

### Surgical explantation or TAV-in-TAV: current perspectives

TAV explantation has been considered as an option for patients with BVF after TAVR. Several retrospective studies have investigated the feasibility and outcomes of this procedure. Hirji et al. in 2020 (20), included 227 patients who underwent TAV explantation, among whom intermediate-risk patients were identified. The thirty-day mortality was at 13.2% and the 1-year mortality rate was 22.9%. Reintervention was mainly performed due to BVF (79.3%). However, infectious endocarditis was responsible for 20.7% of cases, potentially contributing to mortality, although this was not conclusive in subgroup analysis. It's worth noting that patients with endocarditis are beyond the scope of this paper, and surgery remains the primary treatment for them. In the Explant-TAVR registry (21), the one-year mortality rate was at 28.5% with 43.1% of patients presenting with endocarditis of the index TAVR. In 2020, Fukuhara et al. (22) presented data on 15 TAV explantation cases. This study highlighted the surgical challenges of this procedure, including neo-endothelization of the TAV in the aortic wall, which may require endarterectomy and aortic root repair, a challenge less prominent in the surgical valve population. This complexity could be exacerbated with newer generation valves featuring taller sealing skirts as well as with tall-frame TAVR devices. It should be noted that the retrospective nature of the study introduces a selection bias, as high-risk patients would be offered repeat TAVR, while operable patients would undergo surgery. It is also worth discussing that in current reports on TAVR explantation, the reported median time from index TAVR to explantation is around 1 year. These cases might differ from those anticipated in the future. A prolonged duration between the index procedure and the explantation may introduce different failures modes and more challenges with regards to the explantation technique.

TAV explantation appears feasible in operable patients; however, it is associated with high mortality rates and technical surgical difficulties. As a result, TAV-in-TAV has been explored as an alternative option (Table 2). Several registries have investigated the safety of TAV-in-TAV, demonstrating promising results (Table 3). The RedoTAVR (23) and TRANSIT (24) registries reported favorable early and mid-term outcomes, with low rates of mortality, stroke, and coronary obstruction, along with a high rate of device success. The TVT registry (27) showed similar results using only the SEV valve. Nevertheless, these registries included selected patients and anatomies, and the results may not be representative of the entire population eligible for this intervention. The percentage of patients declined due to complex anatomy with a high risk of coronary occlusion was not specified. Nonetheless, TAV-in-TAV remains a safe intervention in selected patients, particularly when comprehensive CT planning is performed. More recently, Makkar et al. (28) published a national registry including 1,320 TAV-in-TAV procedures using balloon expandable valves (BEV). Through propensity score matching, patients were compared with those undergoing native TAVR, revealing low rates of procedural complications and similar mortality rates between both groups. TAV-in-TAV with BEV was deemed a reasonable treatment for failed TAVR for selected patients. Percy et al. (29) conducted a patient-matched analysis comparing TAV-in-TAV to TAV explantation. Their study included 257 patients with TAV-in-TAV and 130 patients with TAV explantation, with a mean age of 76.9 and 75.1 years respectively. Mortality rates after TAV-in-TAV were 6.2% at 30 days and 21% at 1 year, compared to 12.3% and 20.8%, respectively, after TAV explantation. These preliminary findings suggested a potentially lower short-term mortality rate with TAV-in-TAV but similar mid-term results. However, it is important to note several limitations. Patients selected for TAV explantation are, by definition, operable patients, whereas TAV-in-TAV, especially in its early adoption, was considered as an alternative option. Additionally, the study included patients with an index procedure before 2017. Recent generation devices and the lower-risk patients may also influence the outcome of this comparison.

**Table 2:** Criteria favoring TAV-in-TAV vs. TAVR explantation.

	TAV-in-TAV	TAV explantation
Age	✔	✖
Comorbidities	✔	✖
High surgical risk	✔	✖
Risk of patient-prosthesis mismatch	✖	✔
Porcelain aorta	✔	✖
Ascending aortic aneuvrysm	✖	✔
Another concomitant procedure	✖	✔
Anatomical factors		
Annular size (small index valve)	✖	✔
High coronary obstruction risk	✖	✔
Bioprosthetic valve dysfunction type		
SVD	✔	✖
Severe PPM	✖	✔
Severe PVL	✖	✔
Endocarditis	✖	✔
Thrombosis	✖	✔

**Table 3:** TAV-in-TAV registries.

	Redo TAVR registry (23)	Transit registry (24)	Global REDO-TAVI (25)	TVT 2021 (26) Sapien platform	TVT registry 2022 (27) Evolut platform	National registry 2023 (28) BEV
Number of patients	138	172	165	591	292	1,320
Mean age, years	79.2	79.9	80	78.5	78.6	78
STS risk score, %	6.9	6.1	6.83	8.9	10.4	
Indication for TAV-in-TAV	Mainly SVD	SVD	SVD		Mainly SVD	
Index THV failure mode	AS: 37% AR: 29.7% Combined: 32.6%	AS: 33% AR: 56% Combined: 11%	AS: 34.5 AR: 35.2 Combined: 29.7		AS: 44% AR: 62.1%	
Device success, %	85.5	79.7			94.5	
Post procedural mean gradient, mmHg	12.6	10.9		14.6	11.9	
30-day mortality, %	1.4	7	3	4.9	3.2	4.7
1 year mortality, %	11.7	10	11.9	15.6	17.7	17.5
30-day stroke, %	0.7	3.5	0.6	2.4	3.1	2
Coronary obstruction, %	0.7	0	1.2	0.5	0	0.3

More recently, the EXPLANTORREDO-TAVR international registry (30), which retrospectively included 181 patients with TAVR-explant and 215 with TAV-in-TAV, confirmed these results of higher mortality at 30 days for TAVR-explant (13.6% vs. 3.4%) and also at 1 year (32.4% vs. 15.4%), with, however, similar rates on landmark analysis after 30 days.

Patient selection remains pivotal in determining the appropriate treatment. Assessment of a patient's surgical risk and careful evaluation through CT imaging are essential before choosing the best management strategy bearing in mind the importance of considering these aspects at the time of the index TAVR. TAV-in-TAV has shown promising results with fewer complications in selected patients. Future studies should focus on long-term outcomes, anatomical considerations, and patients with low surgical risk.

### TAV-in-TAV: bridging bench studies to clinical expertise

Numerous studies have assessed the feasibility of TAV-in-TAV. From bench studies to clinical experience, efforts have transitioned towards gaining a deeper understanding of the technical aspects of this procedure, pre-procedural planning and its associated challenges.

#### Advancements from bench testing

**Figure 2:**
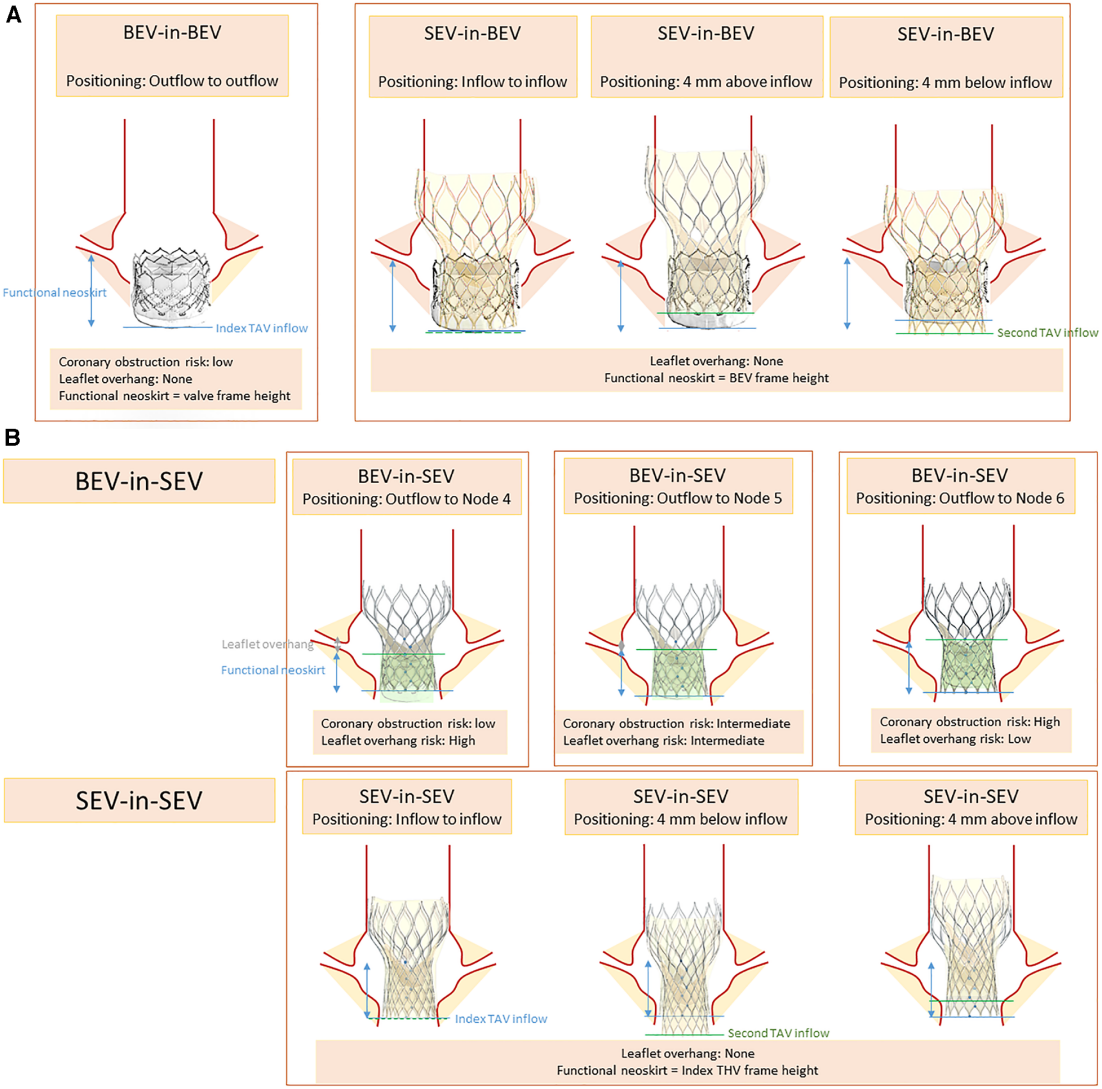
Positioning scenarios and considerations in TAV-in-TAV (**A**) Sapien index THV (**B**) Corevalve index THV.

The TAV-in-TAV implantation technique has been subject to numerous studies. Initially, Sathananthan et al. (31) assessed the performance of various THV models (SAPIEN 3, Evolut PRO, ACURATE neo, ALLEGRA, and Portico). Multiple THV implantation depths were tested and are summarized in Figure 2. A Sapien 3 THV was implanted in a Sapien XT, outflow to outflow. The implantation of a SEV was evaluated at 0, −4, and +4 mm from the inflow of the index THV, while a Sapien 3 was implanted in an evolut R at two positions (high and low). The results were encouraging, demonstrating good hydrodynamic performance for all combinations and positions. However, in a combination of a Sapien 3 implantation in an Evolut R in a high position for small valve sizes, additional inflation volume was required to prevent embolization.

Another important consideration is the depth of THV implantation. The leaflets of the index THV deflect outward against the stent frame, creating a tube graft known as the neoskirt, with the highest point being the top of the trapped leaflets. Akodad et al. (32) studied the depth of a Sapien 3 THV in an Evolut R, by aligning the outflow of the Sapien with nodes 4–6 of the index valve. They found that a lower position reduced the neoskirt of 7.6 mm but resulted in leaflet overhang (ranging from 0% to 94% depending on the THV size). Neoskirt height was lower with short frame valves, which is particularly important when assessing the risk of coronary impairment (33).

Lastly, the phenomenon of leaflets' pinwheeling, characterized by the twisting of the leaflet-free edges due to excessive leaflet redundancy, has been studied. Excessive pinwheeling was observed when implanting a Sapien 3 in a Sapien XT (31). This observation was recently confirmed by Meir et al. (34) who investigated the expansion of TAV-in-TAV using a Sapien 3 (23 mm) in a Sapien XT/3 (23 mm). They found that full valve expansion, which would prevent pinwheeling, was only achieved with pre- and post-dilation. Whether pinwheeling has a long-term impact on valve function remains unknown.

#### Insights from index-TAV CT evaluations

**Figure 3:**
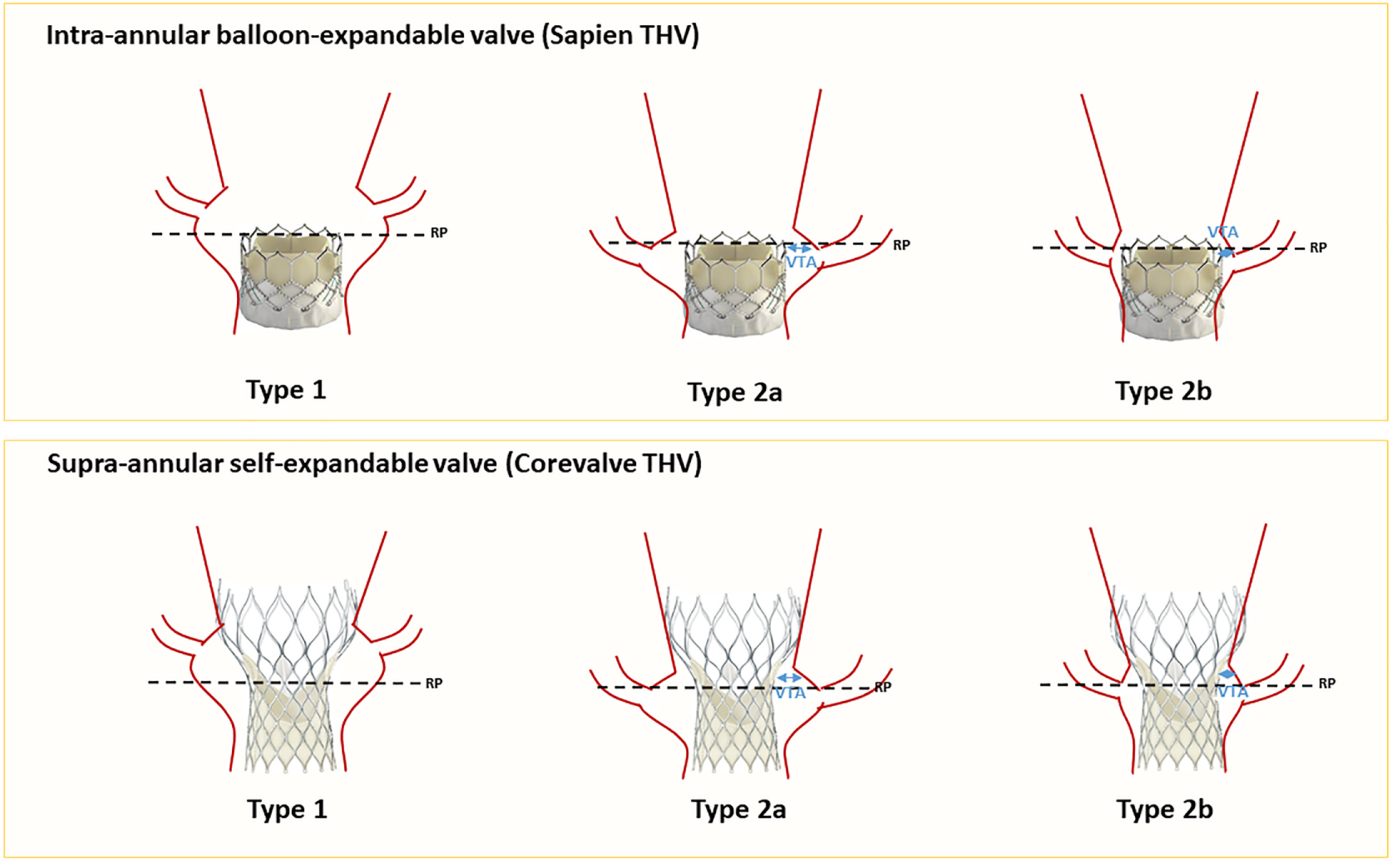
Coronary risk assessment before TAV-in-TAV (for Sapien and Corevalve as index THVs). RP, risk plane; VTA, valve to aorta.

**Figure 4:**
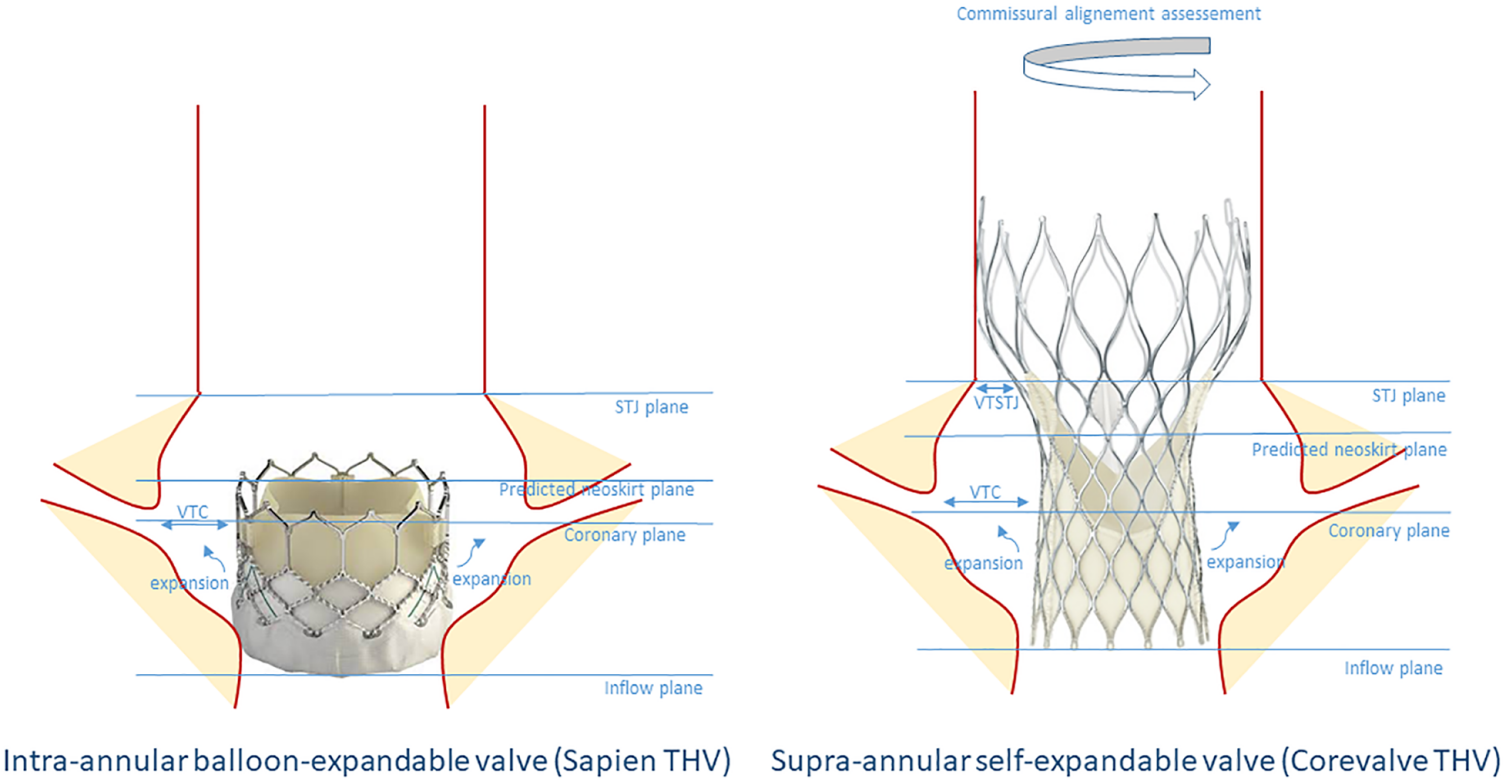
Schematic representation of the criteria to determine the risk of coronary flow compromise before TAV-in-TAV in an index Sapien or Corevalve THV. STJ, sino-tubular junction; VTC, valve to coronary; VTSTJ, valve to sino-tubular junction.

Tarantini et al. (35) introduced an algorithm assessing the risk of coronary flow compromise following TAV-in-TAV based on CT imaging of the index THV. Drawing comparison from the TAV-in-SAV (surgical aortic valve) experience, the authors defined the risk plane (RP) as the level below which the passage of a catheter would be obstructed after implanting a second valve (Figure 3). They described three primary scenarios: Type 1, where the coronary ostium is above the RP; Type 2a where the ostium is below the RP and the valve to aorta (VTA) distance >2 mm and Type 2B where the VTA < 2 mm). This classification enhances the understanding of TAVR feasibility concerning coronary access and potential impairment. Figure 4 describes a representation of these measurements. In a study by Buzatti et al. (36), 221 CT scans following TAVR were analyzed, revealing a high risk of coronary impairment in 55.6% of cases. Beyond the risk of coronary obstruction, there is also a risk of sinus sequestration, which depends not only on the depth of the index THV but also on the measurements of the native annulus (small annulus or short sinus of Valsalva). Conducting a CT scan before the implantation of the index THV may help address challenges associated with native anatomy. More recently, Grubb et al. (37) examined 204 CT scans of patients identified from the Evolut low-risk trial, using an Evolut as the index THV. They simulated TAV-in-TAV procedures with a Sapien 3 targeting inflow-to-inflow implantation, outflow at nodes 4, 5, and 6, as well as an Evolut inflow to inflow. The results indicated that 20% of patients faced a high risk of compromised coronary flow with a Sapien 3 implanted at node 4, compared to 75% at node 6, and 71% for an evolut implantation. Similarly, coronary access was affected in 71% of the Evolut cases, 20% of Sapien 3 cases at node 4, and 75% at node 6. It is noteworthy that there was no coronary obstruction.

#### Enhancing TAV-in-TAV insights through CT analysis

The largest morphological study was conducted by De Backer et al. (38) In their research, they examined 45 TAV-in-TAV patients, comprising 20 Evolut-in-Evolut, 10 Sapien-in-Evolut, 10 Sapien-in-Sapien, and 5 Evolut-in-Sapien cases. They assessed variables such as VTA, valve to coronary (VTC), and strut misalignment. Several key observations were made. Firstly, it was noted that when the first THV used is an Evolut, 90% of the coronary arteries originate below the risk plane, compared to only 6.7% when a Sapien is used as the first THV. Secondly, when the coronary arteries originate below the risk plane, the VTA was less than 2 mm in 27% of cases for Evolut-in-Evolut, 24% for Sapien-in-Evolut, 15% for Sapien-in-Sapien, and 14% for Evolut-in-Sapien procedures. Thirdly, in cases of Evolut-in-Evolut procedures, 10% of patients experienced strut misalignment, potentially leading to inaccessible coronary access. This issue can be mitigated through meticulous commissural alignment during both the index and second procedures (39). When considering all these data, it was found that 27% of patients with an Evolut as the index THV and 10% with a Sapien were considered to have impossible coronary access. These findings may support the selection of an intra-annular valve with a low commissural height and wide cells particularly in younger patients.

#### Insights from clinical practice

Bench studies and investigations based on CT imaging have demonstrated the feasibility of TAV-in-TAV in selected anatomies, contributing significantly to our understanding of this procedure. However, it's important to acknowledge that these data may not fully represent real-world scenarios. The leaflets of the index THV can undergo calcification, potentially affecting the implantation of a second THV. Leaflet overhang, as described when leaflets are not completely trapped between the two valve frames (31), could also impact valve performance, long-term durability, and coronary access. Furthermore, the existing studies often do not encompass the entire patient population, as individuals at risk of coronary obstruction were frequently excluded. Additionally, there is a noticeable absence of large, dedicated studies with robust data, primarily due to the relatively low rates of SVD. Consequently, the medium and long-term outcomes of these patients remain uncertain.

Tarantini et al. (40) published an expert consensus aiming to streamline the TAV-in-TAV procedure, specifically using a BEV. They proposed a tailored step-by-step approach, emphasizing the significance of comprehensive evaluation of native anatomy, commissural alignment of the index THV, the type of SVD, and the risk of coronary obstruction, taking into consideration the reduced space within the sinuses of Valsalva due to valve expansion. In summary, if the index THV is a BEV, they recommend sizing based on the expansion of the initial valve and an outflow-to-outflow positioning. In cases where the index THV is a SEV (such as Evolut), sizing should be based on the inflow and waist of the index THV, accounting for the initial annulus characteristics, and positioning the outflow between nodes 4 and 6, contingent upon the SVD type and coronary risk assessment.

However, it's essential to note that many studies have primarily focused on the Sapien 3 and XT models. Special attention should be given to the Sapien Ultra, which features a higher skirt compared to its predecessors.

#### Procedural recommendations

Several elements guide the TAV-in-TAV procedure. In addition to anatomical data obtained from imaging (VTC, VTSTJ and TAV dimensions), precise assessment of the index TAV is of paramount importance. As previously mentioned, factors such as implantation depth, failure mechanism, and commissural alignment are essential to document. It allows to estimate the anticipated height of the neoskirt. Consequently, certain criteria may favor the implantation of a high-frame THV, such as the Corevalve/Evolut, while other criteria may favor a low-frame THV, such as the Sapien valve. For instance, a Sapien BEV THV might be preferred if there is narrow sinus width, small STJ, or short coronary ostium height, to reduce the risk of sinus sequestration. On the other hand, a high risk of patient-prosthesis mismatch (PPM) or an intervention performed due to PPM could indicate a preference for Evolut as the second valve. The data guiding the choice between these options are summarized in Table 4.

**Table 4:** Criteria favoring BEV vs. SEV as a second THV for TAV-in-TAV.

	BEV (Sapien XT/3)	SEV (Corevalve/Evolut)
Low coronary height	Preferable	Feasible
Small native annular diameter	Feasible	Preferable
Narrow sinuses	Preferable	Feasible
Small STJ/ascending aorta	Preferable	Feasible
Indication		
SVD	Feasible	Feasible
PPM	Feasible	Preferable
Index THV		
BEV/low frame as the index THV	Feasible	Preferable
SEV/High frame as the index THV	Preferable	Feasible
Low implantation depth	Feasible	Preferable

The sizing of the second THV depends on both native anatomy and the index THV. Several elements need consideration, and the following recommendations could serve as a rough guide for common practice:

1. BEV-in-BEV: The same size THV should be used to treat index THV failure (A 20 mm Sapien in a 20 mm Sapien, a 23 in a 23 mm Sapien, a 26 in a 26 mm and a 29 in a 29 mm) and is usually positioned with the outflow at the level of the outflow of the index THV. A lower implantation may be considered if there is a risk of coronary obstruction. The expansion of the second THV depends on the expansion of the index THV, bearing in mind the presence of paravalvular leaks and the risk of coronary obstruction. The additional use of a non-compliant balloon for pre and/or post dilation may be considered to achieve full expansion.
2. BEV-in-SEV: The second THV should be sized to the internal dimensions of the index THV. (Roughly, a 20 mm Sapien in a 23 mm Corevalve/Evolut, a 23 mm Sapien in a 26 mm Corevalve/Evolut, a 26 in a 29 mm and 29 mm in a 34 mm). The position of the BEV THV should be tailored according to the risk of coronary obstruction and the mechanism of SVD (Outflow to node 4–6). While expanding the index SEV with a BEV will cause the Corevalve to remodel outwards, resulting in a gain in the effective orifice area and decreasing the chances of embolization, caution should be exercised regarding the risk of coronary flow impairment which should be taken into consideration.
3. SEV-in-BEV: Sizing should involve using a 23 mm Corevalve/Evolut in a 20 mm Sapien, a 26 mm in a 23 mm Sapien, a 29 in a 26 and a 34 in a 29 mm. Attention should be given to the degree of expansion of the index TAV. The inflow of the second TAV is usually positioned at the level of the inflow of the index TAV.
4. SEV-in-SEV: While this scenario ensures no leaflet overhang, the neoskirt is thought to be the highest with the highest percentage risk of coronary obstruction. A same size valve is recommended, positioned inflow to inflow.

### Unresolved questions

#### Safeguarding against coronary obstruction

Coronary obstruction in TAV-in-TAV procedures is exceptional in published registries, primarily due to careful patient selection. Several factors may contribute to this rare occurrence, including the height of the leaflets, reduced neo-sinuses, and tall valve frames. Tang et al. (41) initially developed a classification system to assess the risk of coronary obstruction before TAV-in-TAV based on the diameter of the sino-tubular junction and the height of the sinuses, categorized into types 1–3. In brief, TAV-in-TAV was considered unfeasible and at high risk of obstruction when the valve-to-sinus distance or valve-to-sinus height was less than 2 mm.

In the context of a Valve-in-valve procedures, various techniques have been introduced to mitigate the risk of coronary impairment, such as the chimney technique and BASILICA (bioprosthetic or native aortic scallop intentional laceration to prevent iatrogenic coronary obstruction). The chimney technique can be employed in a Valve-in valve setting. However, TAV-in-TAV presents the challenge of placing a stent between the metal layers of the THVs, raising questions about the long-term patency of the stent. The use of the BASILICA technique has recently garnered attention. Damlin et al. (42) published a case report describing a successful BASILICA procedure in a TAV-in-TAV setting using a supra-annular device. Nevertheless, it remains uncertain whether this approach is feasible across all anatomies, valve types and sizes. Despite improvements in commissural alignment techniques (43), particularly with the Evolut FX platform (>90% success rate), complete commissural alignment is not achieved in all cases, potentially affecting the feasibility of the leaflet laceration technique. Khan et al. (44) assessed the *in vitro* feasibility of BASILICA in TAV-in-TAV and found that the leaflets of the index THV were less likely to split adequately and could not move beyond the frame of the index THV. This challenge is further compounded with new-generation valves (such as Sapien 3 and Evolut), which exhibited narrower splits after BASILICA compared to earlier models. Greenbaum et al. (45) described the balloon-assisted BASILICA which aims to expand the traversal point in the leaflet with the use of an inflated non-compliant balloon. This technique requires further validation in all valve types and generations. The CATHEDRAL procedure is another procedure that has not been described in a TAV-in-TAV setting (46).

Also, the shortcut device is a device dedicated to splitting the index valve leaflets to prevent coronary obstruction, with precise placement and control of the splitting location. It has been extensively tested in bench test (on surgical valves and THV BEV and SEV) and, preclinical studies. The initial data published on 8 patients showed a complete success in preventing coronary obstruction without stroke. Among these patients, there was a Sapien XT and a Sapien 3 valve (47). An update of this serie was performed at TCT 2023 without the addition of TAV-in-TAV patients. It is important to emphasize the future of this technique in a population where the trend is towards an increase in TAV-in-TAV cases compared to valve-in-valve. Once again, the importance of commissural alignment in the implantation of the index valve is crucial. That said, data pertaining to coronary obstruction prevention techniques remain limited, highlighting the need for further research in this area.

Finally, a hybrid approach could be considered in cases with a high risk of coronary obstruction (SURPLUS TAVR) (48). To avoid a replacement of the aortic root and re-implantation of the coronary arteries, a trans-aortic approach can be performed. This involves a sternotomy and a short aortotomy under extracorporeal circulation. The leaflets of the index valve are resected under the direct visual of the surgeon. Thus, the new valve can be implanted according to standard recommendations, ensuring commissural alignment without the risks associated with the neoskirt. This practice is used in our center for selected cases at high risk of coronary obstruction.

#### Stroke prevention

Despite advancements in TAVR, the rates of stroke have remained stable over the years (49). The risk of stroke following TAV-in-TAV merits discussion. In the study by Hudad et al, the overall stroke rate at 30 days in a large TAVR cohort was 2.3% (49). A meta-analysis comparing stroke rates in TAV-in-SAVR to native TAVR populations showed no significant differences (50). In the TAV-in-TAV population, stroke rates are reported to be approximately 0.5%–3% at 30 days, similar to rates described in TAVR registries. Preventive strategies studied in TAVR populations, such as embolic cerebral protection devices, did not demonstrate an advantage in pre-procedural stroke incidence (51). In the TAV-in-TAV population, the use of these devices is a subject of debate. Studies exploring their utility could be insightful. Certain criteria might guide the operator in identifying the patients that may require the use of these devices including a history of valve thrombosis, extensive calcifications of the index THV leaflets or the necessity of a leaflet modification technique.

#### Post-procedural care considerations

Standardized anti-thrombotic treatment following TAV-in-TAV procedure has not been established. The introduction of foreign material in the TAV-in-TAV setting may increase the risk of leaflet or valve thrombosis. Furthermore, the presence of neosinuses, particularly in low-frame valves, is being recognized as a potential risk factor for thrombosis (52). Future studies should focus on identifying anatomical factors that may necessitate anticoagulation, considering factors such as valve geometries and flow patterns.

### Future perspective

**Figure 5:**
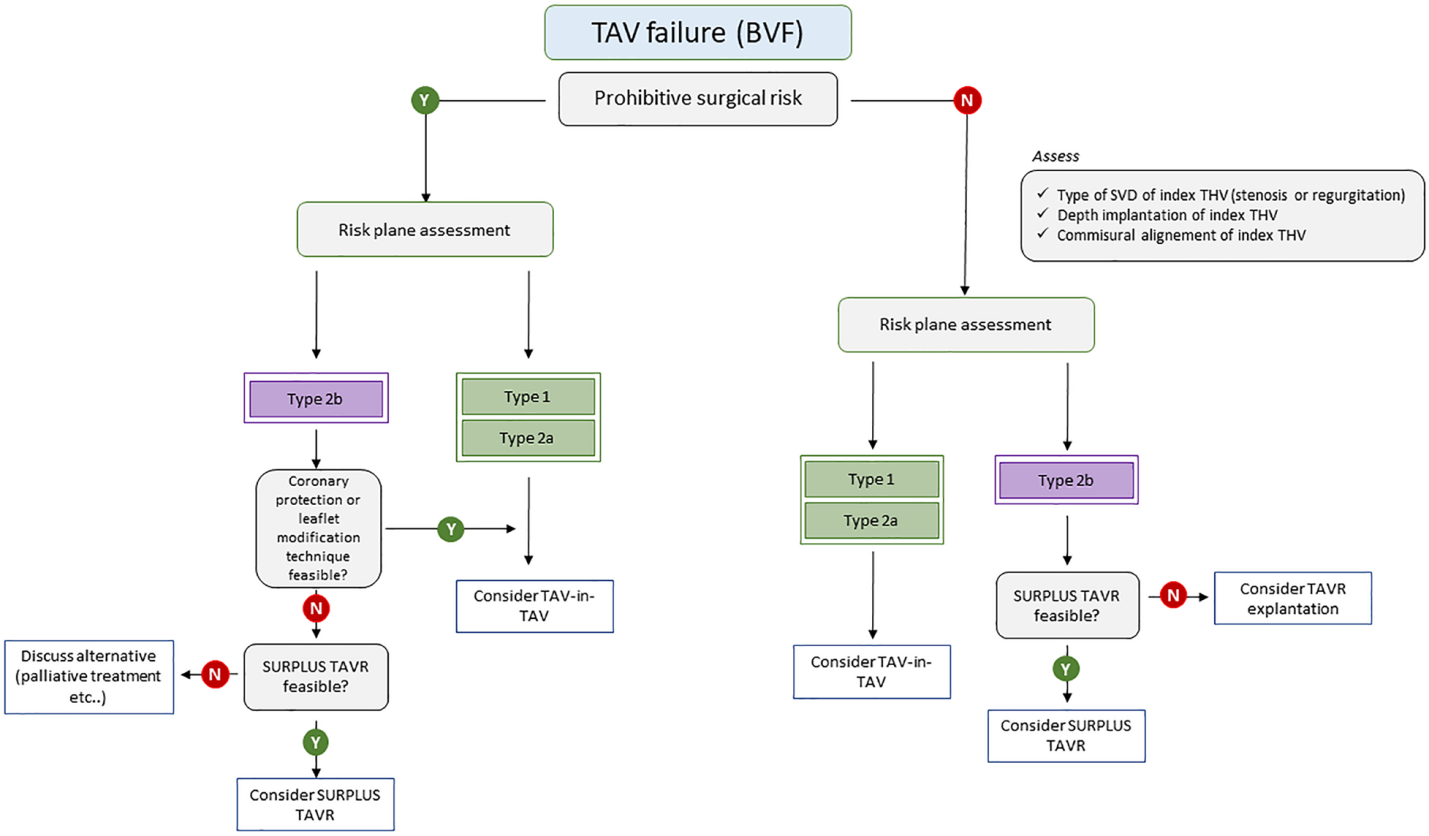
Treatment algorithm of patients with TAV failure. BVF, bioprosthetic valve failure; SVD, structural valve deterioration; TAV, transcatheter aortic valve; THV, transcatheter heart valve.

The future of TAV-in-TAV procedures hold great promise as it enters a phase of exciting development. This technique may become the preferred treatment option for patients experiencing THV failure. Figure 5 suggests a treatment algorithm of patients with TAV failure. To ensure its success, there is a pressing need for more robust evidence and guidelines to guide procedure planning. A heightened focus on assessing the long-term durability, structural integrity, and functional performance of these THVs is on the horizon. This may lead to broader indications, especially as comparative studies between TAVR explantation and TAV-in-TAV in low-risk patients are anticipated to provide essential insights for clinicians. Furthermore, addressing concerns related to the risk of coronary obstruction will be essential to expand the indications of TAV-in-TAV procedures. Above all, a patient-centered approach to lifetime management, starting with the first intervention and considering the likelihood of further interventions, will play a pivotal role in shaping the future landscape of TAV-in-TAV procedures.

## Conclusion

THV durability appears favorable, making TAV-in-TAV an attractive option for patients with TAV dysfunction due to its lower morbidity when compared to TAV explantation. Careful patient selection is paramount, emphasizing the importance of CT assessment and coronary risk evaluation.

Clinical experience is steadily expanding leading to a better understanding of the intricacies of the procedure. Dedicated studies are eagerly awaited to ensure the successful advancement of this technique.
